# Risk profile for drowning deaths in children in the Indian state of Bihar: results from a population-based study

**DOI:** 10.1136/injuryprev-2018-042743

**Published:** 2018-05-19

**Authors:** Rakhi Dandona, G Anil Kumar, Sibin George, Amit Kumar, Lalit Dandona

**Affiliations:** 1 Public Health Foundation of India, Gurugram, India; 2 Institute for Health Metrics and Evaluation, University of Washington, Seattle, Washington, USA

**Keywords:** children, deaths, drowning, India, mortality

## Abstract

**Background:**

We report on incidence of drowning deaths and related contextual factors in children from a population-based study in the Indian state of Bihar which estimated the causes of death using verbal autopsy (VA).

**Methods:**

Interviews were conducted for deaths in 1–14 years population that occurred from January 2012 to March 2014 in 109 689 households (87.1% participation) in 1017 clusters representative of the state. The Population Health Metrics Research Consortium shortened VA questionnaire was used for interview and cause of death was assigned using the SmartVA automated algorithm. The annualised unintentional drowning death incidence, activity prior to drowning, the body of water where drowning death had occurred and contextual information are reported.

**Findings:**

The survey covered 224 077 children aged 1–14 years. Drowning deaths accounted for 7.2%, 12.5% and 5.8% of all deaths in 1–4, 5–9 and 10–14 years age groups, respectively. The adjusted incidence of drowning deaths was 14.3 (95% CI 14.0 to 14.7) per 100 000 children, with it being higher in urban (16.1, 95% CI 14.8 to 17.3) areas. Nearly half of the children drowned in a river (5.9, 95% CI 5.6 to 6.1) followed by in a pond (2.8, 95% CI 2.6 to 2.9). Drowning death incidence was the highest while playing (5.1, 95% CI 4.9 to 5.4) and bathing (4.0, 95% CI 3.8 to 4.2) with the former accounting for more deaths in 1–4 years age group. Sixty per cent of children were already dead when found. None of these deaths were reported to the civil registration system to obtain death certificate.

**Interpretation:**

The findings from this large representative sample of children document the magnitude of and variations in unintentional drowning deaths in Bihar. Urgent targeted drowning interventions are needed to address the risk in children. Gross under-reporting of drowning deaths in children in India needs attention.

## Introduction

Drowning is among the top 10 leading causes of death for children and young adults worldwide, with the drowning death rates at least three times higher in the developing countries than the developed countries.^1 2^ Despite the significant burden, drowning deaths continue to remain an invisible public health issue in most developing countries.^1 3^ In continuing to bring action on drowning, this year, the WHO has released an implementation guide that provides practical steps for preventive measures to address the burden of drowning.^4^

Drowning was reported to be a significant contributor to unintentional injury mortality in under-5 children in India from a large population-based national study done over a decade ago, with a higher incidence reported from the Ganges delta region.^5^ A recent systematic review has highlighted the gaps in the available data to accurately quantify the drowning burden and the need for robust evidence on drowning risk factors to guide prevention initiatives.^6^ Using a previously applied cluster sampling frame,^7 8^ we conducted a population-based study in Bihar state to estimate the causes of death using verbal autopsy (VA). Bihar state is in the Ganges delta region and with a population of 104 million in year 2011, it is the third most populous state in India with 11% of it being urban.^9^ We report drowning death epidemiology for 1–14-year-old population based on VA and highlight the circumstances surrounding the drowning event that could provide a starting point for prevention and intervention opportunities.

## Methods

All participants provided written informed consent; for those who could not read or write, the participant information sheet and consent form were explained by a trained interviewer and a thumb impression obtained.

### Study design

The sampling method is described in detail elsewhere,^7 8 10^ and relevant methods for this report are detailed here. Bihar state is divided into 38 districts each of which is divided into 5–27 blocks giving a total of 342 blocks in the state. Within these 342 blocks, the secondary samplings units (SSUs) were villages in rural areas and urban frame survey blocks in urban areas as defined by the National Sample Survey Organization.^11 12^ The SSUs with <50 households were combined with an adjacent SSU, and the large rural SSUs were split into equal sized segments of 100 households using natural boundaries. A total of 1017 SSUs were sampled in proportion to the number of SSUs in each block, using simple random sampling without replacement. This multistage stratified random sampling approach to obtain a representative sample of 772 rural and 245 urban clusters provided a total of 1017 clusters of about 75–150 households across all the 38 districts of the state of Bihar.

### Data collection

Data collection to enumerate the sampled SSUs was undertaken from June 2014 to July 2015. In each sampled SSU, all the households (a household was defined as people eating from the same kitchen) were enumerated and trained interviewers documented the age and sex of all the usual residents in each household during the period January 2012 and March 2014 using the MS-Access software in hand-held tablets. Details of members who had in/out-migrated, births, deaths during this period were also collected. This exercise identified the households that reported at least one death irrespective of cause between January 2012 and March 2014 for which VA interviews were conducted. The respondent for the interview was that household member >18 years of age who was most aware of the context of death and/or illness preceding death. The Population Health Metrics Research Consortium (PHMRC) shortened VA questionnaire was used for interviews.^13 14^ A direct question documented if the deceased had suffered an injury/accident that led to the child’s death and type and intent of injury were documented. This was followed by recording verbatim an open narrative of the death with the aim to document context around the death. The PHMRC questionnaire was translated into Hindi (local language), after which it was back-translated into English to ensure the accurate and relevant meaning and intent of the questions. Pilot testing of the questionnaire was carried out and modifications made as necessary. Interviews were conducted using the Open Development Kit software in hand-held tablets.

### Data analysis

The cause of death was assigned using the validated SmartVA automated algorithm.^15–17^ The SmartVA was run on all deaths aged 1–14 years and unintentional drowning deaths identified using this run were used in this analysis. Drowning death due to flood disasters and water transport incidents was included. No case of intentional drowning death was reported in this age group. We report the annualised unintentional drowning death rate for the state of Bihar using 2 years data from January 2012 to December 2013 for which population denominator was available. The estimates were adjusted for age, sex and urban–rural distribution for 1–14 years population of Bihar as relevant,^18^ and are reported for 100 000 children. 95% CI are reported for the drowning death rate.

We present detailed descriptive data on drowning deaths using all cases documented from 1 January 2012 to 31 March 2014. We report on the seasonal trends of drowning deaths among children based on the reported date of injury. Two team members (SG and AK) reviewed the open narratives for all drowning death cases and noted the activity prior to drowning and the body of water where drowning death had occurred. Additionally, the open ended narratives were reviewed to highlight contextual information that could allow understanding of prevention and intervention opportunities. Given that the open-ended format of verbatim does not allow computation of numbers, we present case reports across the activities prior to drowning. Other than SmartVA, the rest of the analysis were performed using STATA V.13.1 software (Stata, USA). χ² test is reported where relevant to assess significant associations.

## Results

A total of 224 077 children aged 1–14 years resided in 109 689 households (87.1% participation) between January 2012 to March 2014 (220 244 children in 2012–2013). A total of 58 (54 in 2012–2013) drowning deaths were identified among the 748 deaths this age group. The drowning deaths included one case each of drowning in flood disaster and water transport incident.

### Drowning death rate

**Figure 1:**
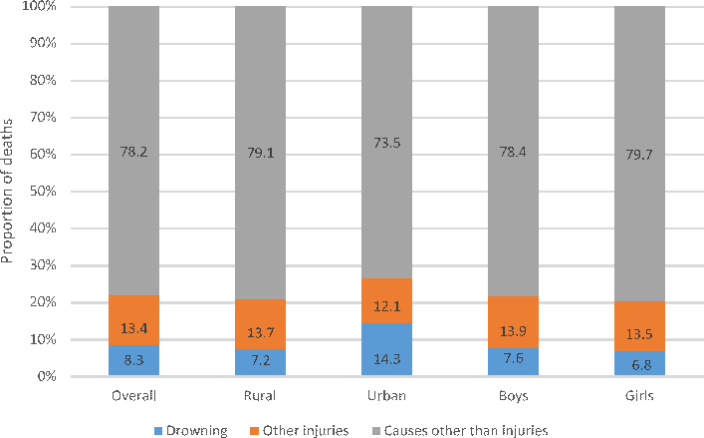
The distribution of causes of death using verbal autopsy interviews by place of residence and sex in children aged 1–14 years in the Indian state of Bihar. Note: Some bars may not add up to 100 because of rounding of decimals.

**Figure 2:**
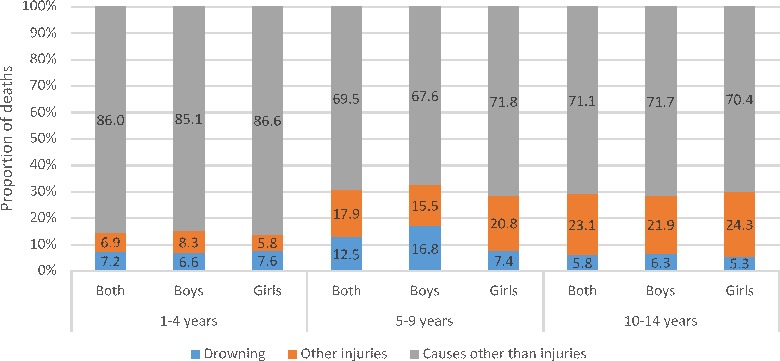
The distribution of causes of death using verbal autopsy interviews by age groups and sex in children aged 1–14 years in the Indian state of Bihar. Note: Some bars may not add up to 100 because of rounding of decimals.

Thirty (51.7%) of the 58 children who had fatally drowned were 1–4 years of age and it accounted for 8.3% of all deaths in this age group (figure 1). The mean (median) age for drowning death was 6.5 (7), 4.9 (3) and 5.8 (5) years for boys, girls and both sexes combined, respectively. Boys in 5–9 years age group (16.8%) had the highest proportion of drowning as the cause of death across the age groups considered (figure 2).

The overall adjusted annualised drowning death rate in 1–14 years children was 14.3 (95% CI 14.0 to 14.7) per 100 000 children, with it being higher in the urban areas than in rural (table 1). The overall adjusted drowning death rate was higher for boys than girls; however, the rate was higher in girls in 1–4 years age group (25.9, 95% CI 24.5 to 27.3) than boys (16.4, 95% CI 15.3 to 17.4) and the latter had a higher deaths than former in the other two age groups (table 1).

**Table 1:** Annualised drowning death rate by sex among children aged 1–14 years based on verbal autopsy interviews in the Indian state of Bihar

Variable	Category	Total sample*		Adjusted drowning death rate per 100 000 children (95% CI)*		
		Boys	Girls	Boys	Girls	Both
Age group (years)	1–4	32 522	30 280	11.5 (10.6 to 12.4)†	25.9 (24.5 to 27.3)†	18.5 (17.7 to 19.3)†‡
	5–9	41 666	38 878	19.7 (18.8 to 20.7)†	8.2 (7.5 to 8.8)†	14.2 (13.6 to 14.8)†‡
	10–14	39 304	37 594	3.4 (3.0 to 3.9)†	2.7 (2.3 to 3.0)†	3.1 (2.8 to 3.4)†‡
Place of residence	Urban	24 287	22 063	22.5 (20.4 to 24.6)§	9.0 (7.6 to 10.3)§	16.1 (14.8 to 17.3)‡§
	Rural	89 205	84 689	10.6 (10.1 to 11.1)§	11.4 (10.9 to 11.9)§	11.0 (10.6 to 11.3)‡§
Overall		113 492	106 752	11.8 (11.3 to 12.2)†§	11.1 (10.7 to 11.6)†§	11.5 (11.1 to 11.8)†‡§

*Sample population and drowning cases considered for 2012–2013.

†Urban–rural adjusted.

‡Sex-adjusted.

§Age-adjusted for Bihar’s population aged 1–14 years.

### Seasonality and body of water

**Figure 3:**
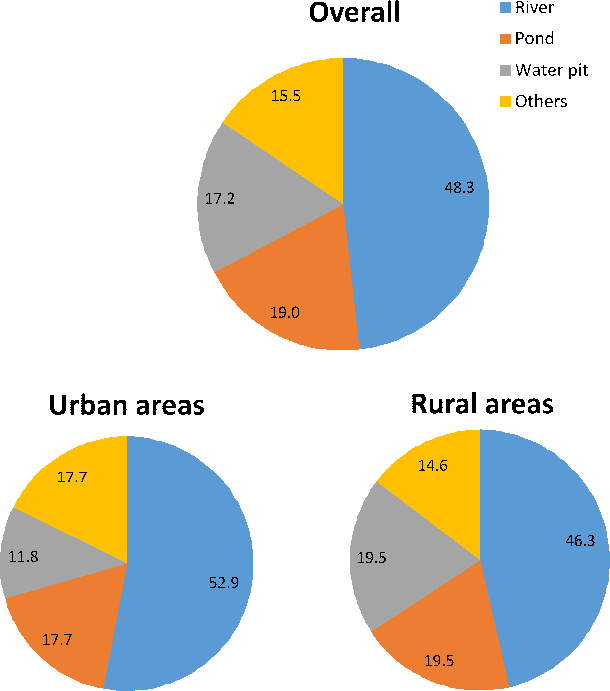
The distribution of drowning deaths by the type of water body in which the children had drowned for children aged 1–14 years in the Indian state of Bihar. Note: Some pie may add more or not add up to 100 because of rounding of decimals.

Thirty-four (58.6%) of the drowning deaths occurred in monsoon season (June–September) followed by 22.4% in summer season (March–May), with similar pattern in urban and rural areas. Almost half of the children drowned in a river (28; 48.3%) followed by pond (11; 19%), water pit (10; 17.2%) and in other water bodies (9; 15.5%) which included wells, canal, tank and sewer (figure 3). Drowning in a river dominated both the urban and rural areas (figure 3). The adjusted annualised drowning death rate in river (table 2) was the highest for 5–9 years age group (10.6, 95% CI 10.1 to 11.1) and for boys (10.2, 95% CI 9.7 to 10.6). On the other hand, adjusted annualised drowning death rate in water pit was the highest in 1–4 years and that of pond in 5–9 years age group (table 2).

**Table 2:** Annualised drowning death rate by type of water body and activity prior to drowning for children aged 1–14 years based on verbal autopsy interviews in the Indian state of Bihar

Variable	Category	Adjusted annualised drowning death rate per 100 000 children (95% CI)*					
		Type of water body†			Type of activity		
		River	Water pit	Pond	Playing	Bathing	Other**
Age group (years)‡§	1–4	7.5 (6.9 to 8.0)	5.2 (4.8 to 5.7)	2.0 (1.7 to 2.2)	14.0 (13.3 to 14.7)	1.6 (1.4 to 1.9)	2.9 (2.5 to 3.2)
	5–9	8.5 (8.0 to 8.9)	0.7 (0.6 to 0.9)	3.9 (3.6 to 4.3)	3.5 (3.2 to 3.8)	6.7 (6.3 to 7.2)	4.0 (3.7 to 4.3)
	10–14	1.8 (1.6 to 2.0)	0.6 (0.4 to 0.7)	0.7 (0.6 to 0.8)	0	3.0 (2.7 to 3.3)	0
Sex‡¶	Boy	6.5 (6.2 to 6.8)	1.0 (0.9 to 1.1)	2.9 (2.7 to 3.2)	3.6 (3.4 to 3.9)	4.9 (4.6 to 5.2)	3.2 (3.5 to 4.0)
	Girl	5.2 (4.8 to 5.5)	2.9 (2.6 to 3.1)	1.5 (1.4 to 1.7)	6.8 (6.4 to 7.1)	3.1 (2.9 to 3.4)	1.3 (1.1 to 1.4)
Place of residence§¶	Urban	9.9 (8.9 to 10.9)	2.0 (1.6 to 2.5)	2.0 (1.6 to 2.5)	5.1 (4.4 to 5.8)	8.7 (7.8 to 9.6)	2.2 (1.7 to 2.6)
	Rural	5.4 (5.2 to 5.7)	2.3 (2.1 to 2.5)	2.8 (2.7 to 3.0)	5.1 (4.8 to 5.4)	3.5 (3.3 to 3.7)	2.3 (2.1 to 2.4)
Overall‡§¶		5.9 (5.6 to 6.1)	2.3 (2.1 to 2.4)	2.8 (2.6 to 2.9)	5.1 (4.9 to 5.4)	4.0 (3.8 to 4.2)	2.3 (2.1 to 2.4)

*Estimates calculated using sample population and drowning cases for 2012–2013.

†Estimates not shown for ‘other water body’ because of small number of cases (7).

‡Urban–rural adjusted.

§Sex-adjusted.

¶Age-adjusted for Bihar’s population aged 1–14 years.

**Includes open defecation, bathing a cow, fishing, rowing a boat, collecting wood and activity not known.

### Activity before drowning

**Figure 4:**
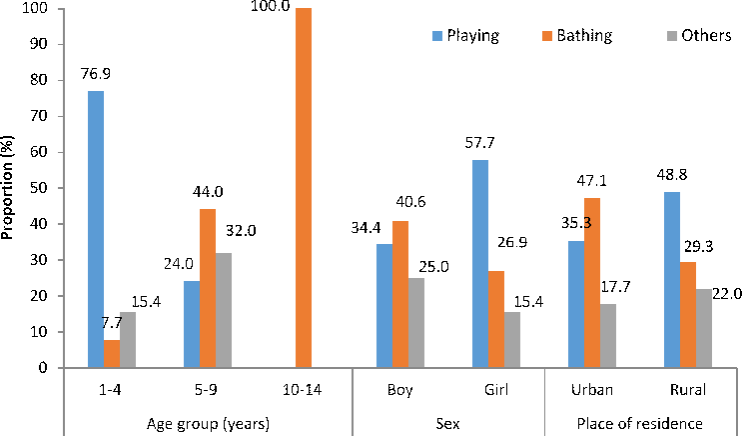
The distribution of drowning deaths by the activity prior to fatal drowning based on age groups, sex and place of residence for children aged 1–14 years in the Indian state of Bihar. Note: Some bars may add to more than 100 because of rounding of decimals.

More children aged 1–4 years drowned while playing near a water body (76.9%) as compared with the other ages (p<0.001), whereas all deaths in 10–14 years age group were during bathing (figure 4). The adjusted drowning death rate while playing (table 2) was the highest for 1–4 years children (16.5, 95% CI 15.8 to 17.3) and that for bathing was the highest in urban residence (8.7, 95% CI 7.8 to 9.6). A higher proportion of girls drowned while playing (15, 57.7%; p=0.076), whereas a higher proportion of boys drowned while taking a bath (13, 40.6%; p=0.275).

### Death and death certificate

Of total 58 deaths, 49 (84.5%) children were already dead when found post drowning and were found alive in 5 (8.6%) cases. In the remaining four (6.9%) cases, it was not clear whether the child was found alive but the child was declared dead on arrival at the health facility. Among the five children who were found alive and died subsequently, four had died on route to a health facility. One child died post multiple referrals on the same day. Overall, in 10 cases (17.2%), access to a health facility was attempted. None of these 58 deaths were reported to the civil registration system to obtain a death certificate.

### Qualitative context review of verbal autopsy narratives

We present case reports by activity before drowning as playing, bathing and open defecation (boxes and 1–3) to highlight the context that led to drowning which could possibly provide guidance for potential interventions to prevent these deaths. The case reports from the VA narratives exemplify the variety of scenarios (translated verbatim as is). The absence of caretaker, lack of attention of the caretaker, no prior knowledge of activity of the child near/in a water body to the caretaker and no knowledge of the child missing to the caretaker are typically recorded in many verbatim.

Box 1 **Case reports highlighting the context for cases in which drowning death had occurred while playing in children 1–14 years of age based on verbal autopsy interviews in the Indian state of Bihar.**

**Case report 1: 2 years old, Respondent: Father**

The child was playing along with other children outside home. When the grandmother went to the hand-pump next to the house, she saw the child in a big bucket with child’s head in the bucket and legs upside down. The child was still slightly breathing when taken out of the bucket and the face was swollen. The child was rushed to the doctor but died on the way. No one knows how and when the child went near the hand pump while playing.

**Case report 2: 1 year old, Respondent: Grandmother**

The child was playing with other children near a septic tank. While playing the child reached near the tank and fell in it because the lid was open. A woman nearby saw this and shouted to gather people. The child was pulled out from the tank. The body was swollen and the child was taken to the doctor who declared the child dead. They brought the child back home for cremation.

**Case report 3: 2 years old, Respondent: Mother**

The mother had taken the child to her parent’s home. There is a river near the home where the child was playing and fell in the river while playing. After 2–3 hours when mother started to look for the child, she found the child in the river. The child was taken out from the water and was dead. The mother felt that the child was thrown in the river by a ghost.

**Case report 4: 1 year old, Respondent: Mother**

The child was learning to walk with the support at that time. One day the child fell into the bucket while playing in the courtyard and was drowned to death in the bucket. Nobody saw how and when the child fell into the bucket.

**Case report 5: 8 years old, Respondent: Mother**

The deceased used to go for extra classes to study. That day also the child went to study. On route, a friend suggested the child to play instead and the child did. Another four children also joined them including the deceased elder brother, and they decided to take bath in River Ganga. All of them returned after bath and waited for the deceased to come out of the river. When the child did not come out, the brother requested the labourers who were having lunch nearby to help him look for the child. They found the child in the river and was still alive. The child was taken to three clinics and all of them referred the child to the big hospital. The doctor in the last clinic gave the child a saline drip. The mother arranged for an ambulance and took the child to the big hospital where the child was declared brought dead.

Box 2 **Case reports highlighting the context for cases in which drowning death had occurred while bathing in children 1–14 years of age based on verbal autopsy interviews in the Indian state of Bihar.**

**Case report 1: 10 years old, Respondent: Father**

On 31 March 2012, the child went with friends for a bath in the river. Suddenly, the child slipped into deep water and started to drown. The friends tried a lot to save the child but were unsuccessful. The child’s body was found after 8 hours.

**Case report 2: 3 years old, Respondent: Neighbour**

The child’s mother went for bath at Gaya Ghat in Patna and took the child with her. While the mother went for bath, the child sat at the shore. However, when the mother finished her bath she could not find her child. After searching, the child’s body was found in the water. The child had drowned.

**Case report 3: 9 years old, Respondent: Father**

A fishing activity was going on in a pond near their village. The child gave an excuse and went there to see it but then decided to take bath in the pond. While bathing, the child drowned. As the child did not return for a while, the family members and villagers started to search for the child who was found in the pond. The child was dead but was still taken to the Darbanga hospital. The doctor declared the child brought dead.

**Case report 4: 14 years old, Respondent: Mother**

The child went with four friends for bathing in the pond. All four went into deep water. The people around the pond saw them drowning and tried to rescue. They were able to pull three of them from the water but they could not find this child. After more than 1 hour of search, the child was found. It was too late by that time as the child had died in the water.

Box 3 **Case reports highlighting the context for cases in which drowning death had occurred while defecating in the open in children 1–14 years of age based on verbal autopsy interviews in the Indian state of Bihar.**

**Case report 1: 6 years old, Respondent: Mother**

On the day of death, the child went to Madrasa for classes. While there, the child went near a pond for defecation. While defecating, the child fell in the pond. The family was not aware of this and had assumed the child to be at class in Madrasa. When the child didn’t return home after the class hours were over, they started to search for the child. They found the child in the pond and was dead.

**Case report 2: 8 years old, Respondent: Mother**

Deceased had gone to attend a wedding at an uncle’s place in Lakhi Sarai. When everyone was asleep in the morning, the deceased went near a pond for defecation. For cleaning after defecation, the child went closer to the pond where the child slipped and fell in the pond. When the family members started to search for the child, they found the child in the pond and was already dead.

**Case report 3: 8 years old, Respondent: Mother**

Respondent said that the child was small and it was rainy season then. The child went to play at 9 o’clock in the morning with other children. While playing, the child went for defecation near the river. In the process of cleaning after defecation, the child’s leg slipped and the child drowned in the river. When the child did not return after the play, the family members started to search. They found the child in the river at around 3 o’clock in the afternoon. The child had drowned to death in the river itself.

## Discussion

The findings from this large representative sample of children documents the variations in unintentional drowning death rates and relative involvement of different bodies of water across the Indian state of Bihar. To our knowledge, these are the first descriptive data from India that provide a starting point for possible actions and for further in-depth investigations of unintentional drowning deaths in addition to highlighting its burden in children.

Data from this study estimate drowning deaths to account for 7.2%, 12.5%, 5.8% and 9.5% of all deaths in 1–4, 5–9, 10–14 and 5–14 years age groups in Bihar, respectively. As the proportion of 1–14 years aged population in Bihar state is 31% higher than the average for India,^18^ these estimates make drowning a substantial burden for children in Bihar. Though drowning deaths occurred throughout childhood, differing profiles were documented by age, sex and type of activity prior to drowning death. Half of the drowning deaths occurred before the fifth year of life.^6^ The 1–4 year olds had drowned mostly while playing and girls accounted for a higher proportion of deaths in this age group. While boys accounted for the most drowning deaths in 5–9 years age group, there was no pattern seen based on type of activity. All children in the 10–14 years age group died while bathing, majority of them were boys. The drowning death rate was higher in urban than in rural areas, particularly for boys. These differing patterns in addition to the context highlighted in the qualitative review of verbatim point to different intervention approaches.^4^ For the younger children, the interventions must focus on parents, caretakers and the home environment, whereas the intervention for older children must focus on children themselves as the drowning occurs when they are without caretakers and alone or with same aged peers in the community.^1 3 4^

Most drowning deaths events in this study occurred in natural water such as rivers and ponds, followed by water pits.^19^ Bihar has eight major rivers and many rural homes use the rivers and ponds as convenient water sources for daily life resulting in children of all ages being exposed to water bodies on a daily basis. This omnipresence of water sources and consequent daily risk of drowning has been highlighted as the single most important determinant of the large difference in drowning rates between the developing and developed countries.^1^ The verbatim review highlighted the predominant activities to be playing and bathing just before these children had fatally drowned. In addition, lack of caretaker or the attention thereof was typically recorded in many verbatim of the younger children. It would seem prudent to use this information to consider options to protect children while engaging in these activities near/in a water body or restrain access to water. Installing barriers to control access to water, provision of safe places for children to play with a capable caretaker and teaching school-aged children basic swimming and water rescue skills are recommended.^1 4 20^ While we did not document the ability to swim in the deceased, it is widely known that children in rural areas in India learn to swim early in childhood from peers and family given the proximity to water bodies. This also corroborates with documentation of a significantly higher drowning deaths while bathing in urban children than in rural in our study. Children in urban areas are not routinely exposed to swimming and it is also not typically taught in most Indian schools; access to swimming pools is generally very low in India. It is also important to point out that in addition to the natural waterbodies, the qualitative data also documented drowning deaths in buckets and water storage containers placing the young children at risk. Drowning death of a child post open defecation during the process of cleaning him/herself in the water body deserves a mention as open defecation is still practiced widely in India.^21^ Various adverse health outcomes related to open defecation have been documented previously,^22–24^ but not drowning death. As only 25.2% of Bihar’s households have access to improved sanitation, reducing open defecation is even more important given the drowning risk for children.^25^

Most of the children were found dead after the drowning event had occurred, which is similar to reports from elsewhere.^1 4 20^ As the verbatim was unstructured, the exact time between the drowning event and someone becoming aware of the event and of duration of submersion in water were not available. However, most verbatim suggested a delay between drowning and finding the body of the drowned child. This delay, as suggested by the verbatim, was mostly the delay in the knowing the whereabouts of the child or knowledge about the drowning event per se to the caretaker, family or friends. A recent meta-analyses of outcomes of drowning at scene concluded that a shorter submersion period and a shorter emergency response time or prompt resuscitation was associated with favourable outcome.^26^ In the context of our study findings, this translates into increased supervision of children, reduced time gap between drowning and its knowledge to caretakers and the need for bystander resuscitation which is also highlighted by the less number of children who were found alive but did not survive to reach a health facility.^27 28^ Drowning is a leading cause of death for all age groups of children after infancy globally including from the countries in the South East Asia region—Bangladesh, Cambodia, China, Thailand and Viet Nam.^3^ Similar risk factors for drowning deaths as in this study have been documented in these countries and in India.^1 3 6^ The developed countries have largely eliminated hazardous environment by building a culture of water safety.^3 20^ Because of the different reality and context of less developed countries, not all interventions from the developed countries are relevant or directly applicable to the less developed countries. Until recently, there was a lack of well-designed drowning death prevention trials in developing country setting.^20 29–31^ Recent large-scale drowning prevention trials in Bangladesh, a neighbouring country with a high burden of drowning deaths in children,^32–34^ including a village crèche programme and a programme to teach swimming, water safety and water rescue skills, documented large and significant reduction in drowning deaths.^20 29 35 36^ Given the similar epidemiology and risk factors in Bihar and Bangladesh, there are certainly lessons to be learnt for immediate implementation of such programmes with relevant local adaptation and also about the challenges for implementing drowning interventions in developing countries.^29^

With eight river basins in Bihar, it is one of the flood-prone states in India.^37 38^ The death toll from the recent floods in 2017 was at 514, the highest in the last 9 years in Bihar.^39^ Drowning deaths get recognition when deaths due to a natural disaster such as flooding or due to boat/ferry sinking are reported by the media,^40–42^ thereby, creating an incorrect impression that such situations cause most drowning.^3^ Individual drowning deaths are rarely reported. Extrapolating the data from our study, an estimated 5400 children 1–14 years died in Bihar in 2016 due to drowning, 10 times more than the recent floods toll for all ages. Furthermore, none of the drowning deaths in our study were reported to the civil registration system to obtain a death certificate and hence reflect the gross undercount in the national statistics for drowning deaths.^6 43^ This undercount has implications for highlighting drowning deaths as a public health issue and it also adds to the undercount of fatal injury statistics, thereby, adding to the continued neglect of injuries.^44^ Given this undercount of drowning deaths in the current system for determining and reporting drowning deaths, there is an urgent need to count deaths with reasonable accuracy for informing health policy and resource-allocation decisions.

There are several strengths of this study. A large representative sample of children from a well-defined population, deaths over a 2-year period and details of antecedent events and risk factors. A limitation is that because this study was designed to capture all causes of death and not specifically the context or risk factors for drowning deaths per se, we have used the information available from the open verbatim to present antecedent events and risk factors. We believe that the verbatim captured the primary activity as known to the respondent that the child was engaged in when drowning death occurred, but it is also likely that a child could have been involved in more than one activity at that time such as playing while taking bath. Despite the limitations, we believe that the useful information provided by this study is a starting-point for action and for further in-depth investigations. Overall, these data add to increase the visibility of drowning deaths by addressing many of the gaps in magnitude and risk factors for drowning deaths among children in India.

In conclusion, drowning interventions are currently not included in child mortality reduction interventions in India.^45^ These data suggest that the drowning interventions for children in Bihar are needed and have to begin early if it were to be effective in addressing the children at the risk of drowning deaths. Such interventions will add to achieving the Sustainable Development Goal three to reduce under-5 mortality for all causes of deaths.^46^

**What is already known on the subject**

- Drowning is among the 10 leading causes of death for children and young adults worldwide.
- Drowning was reported as a significant contributor to unintentional injury mortality in under-5 children in India from one large population-based national study done a decade ago.
- Limited data are available on drowning deaths and risk factors to accurately quantify the drowning burden and to guide prevention initiatives in India.

**What this study adds**

- This study provides current population-based estimates of drowning deaths in children from one of the most populous Indian states using verbal autopsy. In 1–14 years age group, drowning deaths accounted for 8.3% of all deaths in the state and half of the drowning deaths occurred before the fifth year of life.
- Though drowning deaths occurred throughout childhood, differing profiles were documented by age, sex and type of activity prior to drowning. Contextual information is provided as case-reports which can provide a starting point for prevention and intervention opportunities.
- Gross under-reporting of drowning deaths in children in the national statistics is documented.
